# The Surgical Anatomy of Inguinal Herniæ, the Testis and Its Coverings

**Published:** 1841-10

**Authors:** 


### PART SECOND.

### Bibliographical Notices.

---

ART. I.—*The Surgical Anatomy of Inguinal Herniæ, the Testis and its Coverings.* By THOMAS MORTON, one of the Demonstrators of Anatomy in University College, London. *Illustrated with Lithographic Plates and Wood Engravings.—London,* 1841. 8vo, pp. 330.

WE have here another production from the industrious pen and pencil of Mr. Morton. The object of the author is to give an accurate anatomical description of the parts he treats of, and to combine with it all the surgical information which can be brought to bear upon the subject. We willingly yield to Mr. Morton all the credit due to the successful accomplishment of the object proposed.

In a work like the present we do not look for anything novel in a surgical point of view: but we have a careful selection of orthodox opinions from the writings of our most approved surgical authorities, on which, therefore, the student may rely.

In the anatomical division of the work we have noticed some slight inaccuracies, which are probably oversights. Thus, in describing the dartos, Mr. Morton speaks of it as a modification of the superficial fascia of the abdomen, "upon which the vermicular movements and gradual contractions of the scrotum are dependent." (pp. 214-5.) The dartos is a structure *sui generis,* and completely distinct from the superficial fascia: and the corrugation of the scrotal integument is alone referrible to the contraction of the dartos, whilst the vermicular movements of the testis are doubtless dependent on the spontaneous action of the cremaster.—Again, it is not an usual arrangement of the fibres of the intercolumnar fascia, that "near the apex of the external ring, they are disposed with their *concavity* downwards towards the ring." (p. 222.) Mr. Morton here confuses the intercolumnar bands with the fascia of the external ring: the arrangement he speaks of would materially interfere with the purpose which the former structure is destined to answer.—The existence of *one,* instead of two, epigastric veins, we consider the exception to the rule. (p. 253.) These, however, are trifling defects in an excellent work.

The present volume contains many engravings laboriously coloured, and some woodcuts: we cannot help repeating the preference we give to the latter. In conclusion we must in justice state that the student who wishes for *assistance* in the acquirement of his surgical anatomy, will find a safe guide in the work before us: but let him beware of the temptation which is held out to him of resting satisfied without consulting the book of nature for himself. We are sure Mr. Morton is the last man to counsel acquiescence in such a plan of study.

